# Occult Ductal Carcinoma In Situ Presenting as Benign Papilloma: A Case of Diagnostic Evasion in Bloody Nipple Discharge

**DOI:** 10.7759/cureus.90959

**Published:** 2025-08-25

**Authors:** Muhammad Hamza Shah, Mushahida Batool

**Affiliations:** 1 School of Medicine, Queen's University Belfast, Belfast, GBR; 2 Internal Medicine, Antrim Area Hospital, Antrim, GBR; 3 Centre for Anatomy, The University of Edinburgh, Edinburgh, GBR; 4 Breast Surgery, Omar Hospital & Cardiac Centre, Lahore, PAK

**Keywords:** malignancy risk, nipple discharge, papillary dcis, surgical intervention, women’s health

## Abstract

Pathologic nipple discharge in postmenopausal women is a clinical red flag that may conceal underlying malignancy, most notably ductal carcinoma in situ (DCIS), despite often being attributed to benign intraductal papillomas. We report the case of a 59-year-old woman presenting with spontaneous, unilateral bloody nipple discharge. Imaging identified a retroareolar hypoechoic lesion without microcalcifications, suggestive of a benign papilloma, and core needle biopsy revealed no atypia or malignancy. However, due to persistent symptoms and radiopathological discordance, the patient underwent surgical duct excision. Histopathology demonstrated DCIS of cribriform and micropapillary subtypes, confined to the ducts with clear margins. This case highlights the limitations of imaging and biopsy in detecting occult carcinoma within papillary lesions and reinforces the role of surgical excision as the gold standard for both diagnosis and treatment in patients with symptomatic nipple discharge.

## Introduction

Pathologic nipple discharge, defined as spontaneous, unilateral discharge from a single duct, especially if bloody or serosanguinous, is a well-recognized warning sign of underlying breast disease [1]. While the majority of such cases are ultimately due to benign causes (with intraductal papillomas being the most common lesion identified), a significant minority harbor malignancies. Published series have reported that roughly 5-12% of women presenting with suspicious nipple discharge will be diagnosed with breast carcinoma, most often ductal carcinoma in situ (DCIS) [2]. Indeed, DCIS is the single most common malignancy associated with pathologic nipple discharge, even though it is more typically detected via screening mammographic microcalcifications rather than clinical symptoms [3].

This means that an occult in-situ carcinoma can occasionally manifest with nipple discharge despite minimal findings on routine imaging. Conversely, most benign papillomas causing discharge are not associated with calcifications, and therefore distinguishing a benign papillary lesion from early DCIS on imaging can be extremely challenging in these patients [4]. Diagnosing the exact cause of bloody nipple discharge often requires a combination of imaging and tissue sampling, but even then, pitfalls remain. Core needle biopsy of an intraductal papilloma may yield benign histology yet under-sample an adjacent focus of atypia or DCIS, leading to a false sense of security. It has long been recognized that papillary lesions identified on core biopsy carry a risk of “upgrade” to malignancy upon surgical excision; studies have documented upgrade rates of about 8-12%, which is why the historical recommendation was to excise all intraductal papillomas [5,6]. More recent data suggest that truly asymptomatic papillomas without atypia (with complete radiologic-pathologic concordance) have a much lower malignancy rate, on the order of only 1-5%, and can be observed with close follow-up in select cases [7]. Clinical context, however, is paramount. Papillomas that present with concerning symptoms (e.g., spontaneous bloody discharge or a palpable mass in an older patient) are associated with a higher likelihood of underlying DCIS or invasive cancer, even if the core biopsy is benign [4]. Thus, the current best practice is to maintain a low threshold for surgical excision in any patient with pathologic nipple discharge and discordant or indeterminate findings. This approach ensures that an occult malignancy is not missed and provides both definitive diagnosis and treatment.

Here we report a case of a 59-year-old postmenopausal woman with unilateral bloody nipple discharge in whom initial work-up suggested a benign intraductal papilloma. Despite benign core biopsy findings, the persistence of symptoms prompted surgical excision, which ultimately revealed “occult” DCIS within the resected ducts. This case highlights the diagnostic challenges in evaluating nipple discharge and emphasizes the importance of correlating clinical, radiologic, and pathologic findings to avoid a missed breast cancer diagnosis.

## Case presentation

A 59-year-old postmenopausal woman was referred to the symptomatic breast clinic with a two-month history of spontaneous, unilateral bloody nipple discharge from the left breast. The discharge was described as bright red, intermittent, and not associated with any breast pain, palpable mass, nipple inversion, or skin changes. There was no history of trauma, infection, or recent hormonal therapy. Her gynecological and obstetric history was unremarkable. She reported no prior breast interventions or surgeries and had never undergone mammographic screening. There was no significant family history of breast or ovarian cancer, and she had no known BRCA mutations. On clinical breast examination, the breast was soft and symmetrical with no palpable masses, skin tethering, peau d’orange, or nipple retraction. However, bloody discharge was readily reproducible from a single duct upon gentle manual expression of the left nipple. The contralateral breast and axillary lymph nodes were unremarkable.

**Figure 1:**
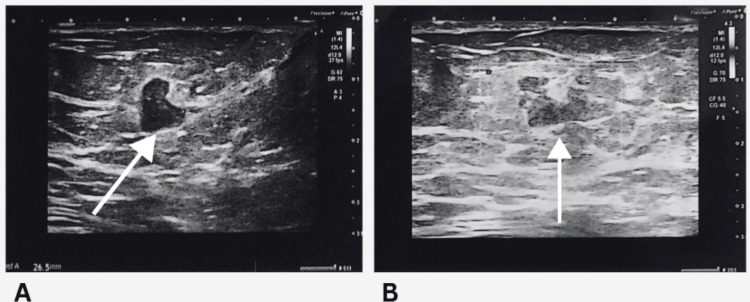
Ultrasound images showing a hypoechoic lesion within a dilated duct, suggestive of intraductal papilloma versus low-grade DCIS. (A) Longitudinal view revealing a well-defined, lobulated hypoechoic lesion (arrow) within a markedly dilated duct. (B) Transverse view showing the same hypoechoic lesion (arrow) embedded in heterogeneous surrounding parenchyma, consistent with ductal involvement. DCIS: ductal carcinoma in situ.

As part of the standard triple assessment, the patient underwent diagnostic imaging. Mammography revealed a subtle, ill-defined density with an associated hypoechoic lesion located in the retroareolar region of the left breast. No microcalcifications or architectural distortions were seen. This lesion was categorized as BIRADS category III to IV, indicating a finding suspicious for but not definitively diagnostic of malignancy. A targeted breast ultrasound corroborated the mammographic findings, identifying a small, well-circumscribed hypoechoic lesion within a dilated duct, raising the differential diagnosis of an intraductal papilloma versus a low-grade intraductal carcinoma (Figure 1).

**Figure 2:**
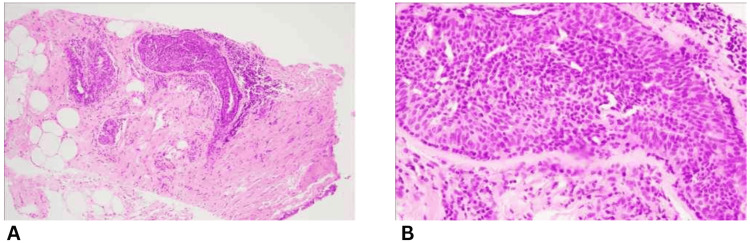
Core needle biopsy showing benign breast parenchyma with stromal sclerosis and usual ductal hyperplasia. (A) Hematoxylin and eosin (H&E) stain, low-power view (4× magnification) demonstrating preserved ductal-lobular architecture with areas of stromal fibrosis and ductal epithelial proliferation. (B) H&E stain, high-power view (40× magnification) highlighting usual ductal hyperplasia composed of uniform, bland epithelial cells with an intact myoepithelial layer.

Given the radiological findings and ongoing discharge, an ultrasound-guided core needle biopsy (trucut) of the lesion was performed. Multiple core tissue samples were obtained under aseptic conditions and submitted in formalin for histopathological analysis. Microscopically, the cores showed benign breast parenchyma with areas of stromal sclerosis, mild chronic inflammatory infiltrate, and usual ductal hyperplasia (Figure 2). Importantly, there was no histological evidence of cellular atypia, architectural distortion, or malignancy. Immunohistochemical staining demonstrated intact myoepithelial layers (p63-positive) and patchy estrogen receptor (ER) positivity, both features consistent with a benign lesion.

Despite the benign histological report, the discordance between the radiological suspicion and pathological findings raised concern for sampling error or underestimation of the lesion. The persistence of bloody discharge, particularly in a postmenopausal woman, and the localization to a single duct further heightened suspicion. Multidisciplinary team (MDT) discussion recommended definitive surgical excision to obtain a more representative tissue sample and provide symptom relief. The patient underwent a Hadfield’s procedure, involving targeted excision of the affected ductal system. Intraoperatively, the duct was noted to be papillomatous and friable, but a section was thickened and fibrotic, adherent to surrounding subareolar tissue. Both suspicious ducts were excised en bloc, and the specimen was sent for histopathological evaluation.

Gross examination of the excised tissue revealed ductal structures with focal intraluminal papillary projections. Microscopic analysis of the ductal tissue demonstrated features consistent with DCIS, predominantly of the cribriform and micropapillary subtypes, with low to intermediate nuclear grade and focal necrosis. The lesion was confined within the ductal system, with no evidence of stromal invasion. Immunohistochemistry confirmed the absence of myoepithelial cells surrounding the involved ducts (p63-negative), and ER expression was diffusely positive. No invasive carcinoma or lymphovascular invasion was identified, and surgical margins were clear.

## Discussion

This case highlights the diagnostic challenges of differentiating benign papillary lesions from early breast carcinoma in patients with pathologic nipple discharge. Bloody, unilateral, single-duct nipple discharge in a postmenopausal woman is a red-flag symptom, as a significant minority of such cases conceal an underlying malignancy. In DCIS, nipple discharge results from malignant cells proliferating within the ducts, and although DCIS is often detected via mammographic microcalcifications, it may sometimes manifest clinically with discharge or a subtle lump [8].

In our patient, imaging raised suspicion by revealing a small retroareolar lesion, but notably there were no microcalcifications. DCIS typically appears as fine, linear, or branching calcifications on mammograms and only less frequently as a mass or asymmetry [8]. The absence of calcifications in this case likely contributed to the lesion’s “occult” nature, making it more difficult to characterize definitively on imaging. Ultrasound did identify a dilated duct with a lesion, consistent with an intraductal papilloma versus papillary carcinoma. However, imaging alone cannot reliably distinguish benign from malignant papillomas in many instances. Even in experienced series, mammography and ultrasound can miss or underestimate the extent of malignancy in nipple discharge patients, highlighting the importance of pathological evaluation [9]. In this context, our patient’s core needle biopsy, initially showing only benign changes (usual ductal hyperplasia and inflammation), was discordant with the radiologic and clinical picture. This discordance raised the concern of a sampling error or an “underestimation” of the lesion’s pathology, which ultimately proved to be the case. False-negative biopsies of papillary breast lesions are a well-recognized pitfall. Papillomas within ducts can be heterogeneous, with areas of both benign papilloma and adjacent foci of atypia or DCIS [10]. A core needle biopsy samples only a portion of the lesion, so malignancy can be missed if the sample does not capture the aggressive focus. In fact, the management of intraductal papillomas diagnosed on core biopsy remains controversial. Despite conflicting data, a few studies suggest that close imaging surveillance may be acceptable for incidentally discovered, asymptomatic papillomas that show no atypical features.

Importantly, however, the presence of clinical symptoms (especially nipple discharge or a palpable mass) and other risk factors significantly increases the odds that a papilloma may harbor an occult carcinoma. In one large series, papillomas presenting with symptoms were far more likely to be “upgraded” to DCIS or atypia on final surgery than asymptomatic lesions [11]. Consistent with this, our patient’s persistent, spontaneous bloody discharge (combined with imaging suspicion) warranted further intervention despite the benign core biopsy. Many breast specialists err on the side of caution in such cases: thus, current best practice is to pursue surgical excision for any papillomatous lesion with discordant findings or worrisome clinical features. Surgical excision (e.g., a microdochectomy/Hadfield’s procedure targeting the affected duct) remains the gold standard for both diagnosis and treatment of pathologic nipple discharge.

Major duct excision not only provides symptom relief but also yields a larger, contiguous tissue specimen for thorough histopathological examination [12]. In our case, excisional biopsy was decisive: it revealed DCIS of the breast that had evaded detection on the smaller core samples. The DCIS was of predominantly cribriform and micropapillary subtypes, low-to-intermediate nuclear grade, confined within the ducts. Notably, micropapillary DCIS is an uncommon variant known to be associated with more extensive or multifocal disease within the ductal system [13]. This histologic pattern can architecturally mimic a benign papilloma (with delicate papillary fronds) but lacks the true fibrovascular cores and myoepithelial layer of a benign papilloma. In our patient’s excised specimen, immunohistochemistry confirmed the absence of myoepithelial cells around the involved ducts (p63-negative), verifying the in situ carcinoma. Had the lesion not been excised, this DCIS could have persisted or progressed to invasive cancer over time. Fortunately, the surgical margins were clear and no invasive component was identified, indicating that the disease was caught and removed at an early stage.

This case outlines several key points for clinical practice. First, in an older patient, unilateral bloody nipple discharge should be approached with a high index of suspicion for malignancy, even if initial tests are reassuring. Second, radiologic-pathologic concordance is critical: a benign biopsy should not be accepted at face value when imaging suggests otherwise. Third, complete excision of the involved duct(s) remains the definitive diagnostic step in cases of persistent pathologic discharge, as even advanced imaging (ductography or MRI) can miss small intraductal tumors. Finally, this case adds to the literature on “occult” DCIS arising within what initially appeared to be a benign papilloma. Similar reports document patients in whom DCIS was only discovered upon excisional biopsy of a papillomatous lesion. In summary, our experience highlights the need for vigilance and a low threshold for surgical evaluation in patients with suspicious nipple discharge. By adhering to thorough work-up and not dismissing discordant findings, clinicians can avoid diagnostic delay and ensure that early breast cancers such as DCIS are appropriately identified and treated.

## Conclusions

This case illustrates how DCIS can masquerade as a benign papilloma, evading diagnosis on imaging and core biopsy. In postmenopausal women with unilateral bloody nipple discharge, persistent symptoms and radiopathological discordance should prompt surgical excision, even in the absence of overt malignancy on initial workup. Microdochectomy not only provided definitive diagnosis in this case but also facilitated timely intervention. Clinicians should maintain a low threshold for excision in similar presentations to avoid delayed diagnosis of early breast cancer.
